# Bidentate [C,N] and Tridentate [C,N,S] Palladium Cyclometallated Complexes as Pre‐Catalysts in Cross‐Coupling Reactions

**DOI:** 10.1002/open.202400253

**Published:** 2024-11-29

**Authors:** Basma al Janabi, Francisco Reigosa, Juan M. Ortigueira, José M. Vila

**Affiliations:** ^1^ Departamento de Química Inorgánica Universidad de Santiago de Compostela, E- 15782 Santiago de Compostela Spain

**Keywords:** Palladium, Schiff bases, Phosphines, X-ray diffraction, Catalysis

## Abstract

Treatment of halide substituted bidentate [C,N] and tridentate [C,N,S] Schiff base ligands with palladium(II) acetate or with lithium tetrachloropalladate, as appropriate, afforded through C−H activation of the organic ligand, the dinuclear (**1 a**, **1 b**) and mononuclear (**1 c**, **1 d**, **1 e**) palladacycles, respectively. Reaction of the μ‐acetate dinuclear complexes with aqueous sodium chloride in a metathesis process, gave the μ‐chloride dinuclear complexes (**2 a**, **2 b**). Treatment of the latter with triphenylphosphine or with bis(diphenylphosphino)methane (dppm)/ammonium hexafluorophosphate in complex/ phosphine 1 : 2 ratio, gave the phosphine derivatives (**3 a**, **3 b**, **4 a**, **4 b**). Reaction of **1 c** and of **1 e** with triphenylphosphine/silver perchlorate and dppm/silver perchlorate, respectively, gave compounds **2 c** and **2 e** with removal of the chloride ligand by precipitation of the silver halide. The compounds were characterized by microanalysis (CHN), IR and ^1^H and ^31^P{^1^H} NMR spectroscopy. The crystal structures of three complexes, **4 a**, **1 c** and **1 e** were studied by single‐crystal X‐ray diffraction. Furthermore, the performance of the palladacycles were tested as pre‐catalysts, namely Suzuki‐Miyaura cross‐coupling reaction and those furnishing the better results are also reported.

## Graphical abstract

Substituted bidentate [C,N] and tridentate [C,N,S] Schiff base ligands and palladium(II) acetate or lithium tetrachloropalladate, as appropriate, afford by C−H activation of the organic ligand, dinuclear and mononuclear palladacycles, respectively. These complexes and the resulting phosphine derivatives are tested as precatalysts for the Suzuki‐Miyaura cross‐coupling reaction.

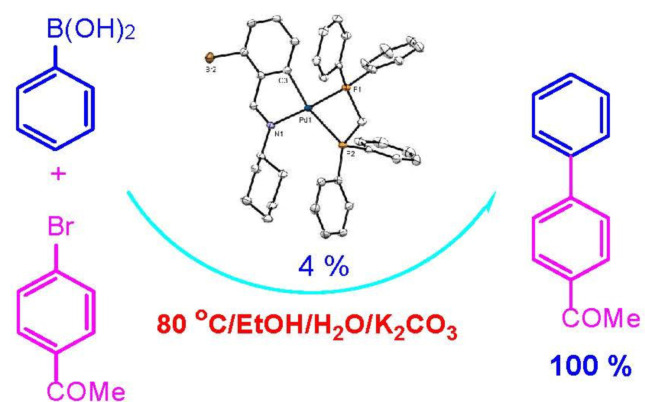

## Introduction

The C−H activation of organic ligand which led to the cyclometallation reaction was first reported by Cope and Siekman[^1^, ^2^, ^3^, ^4^] Since then, the activation of aromatic C‐ H bonds by transition metals has become a most important part of organometallic chemistry. Quite many of metals, especially transition metals, and of organic ligands have been used since then. Noteworthy among the former are palladium^[5]^ and platinum,^[6]^ and as for the latter imines,^[7]^ thiosemicarbazones,^[8]^ and pincer ligands^[9]^ should be highlighted. In the special case of the palladacycles their structural characteristics, their reactivity patterns and also the numerous applications they show have led them to meet the highest standards of research. Some important ones are their use as phosphorescent compounds,^[10]^ metallomesogens,[^11^, ^12^] as antineoplastic species[^13^, ^14^, ^15^] in synthetic chemistry to functionalize aromatic carbon atoms.^[16]^ In catalysis Hermann *et al*.[^17^, ^18^] first used palladacycles with phosphorus donor atoms within the metallacycle ring. Also, palladacycles have been employed in a wide variety of cross‐coupling reactions: Suzuki–Miyaura,[^19^, ^20^, ^21^, ^22^] Mizoroki–Heck,[^23^, ^24^, ^25^, ^26^] and others.^[27]^ The Schiff base ligands initially made by H. Schiff^[28]^ are highly prone to produce palladacycles; they are readily synthesized and the starting materials, amines and aldehydes, are rather numerous more often than not, low cost reagents. For the ligands bearing aromatic ring the metallation site may be tuned by convenient changes in the substituents on the ring.^[23]^ The ensuing palladacycles act as pre‐catalysts that deliver the correct amount of Pd(0), or they may be intermediates; in any case they are the choice complexes for the Suzuki‐Miyaura cross‐coupling.^[23]^ We suggested that the tridentate [C,N,S] ligands would give less active catalysts in view of our previous work related to tridentate Schiff bases and thiosemicarbazones due to the fact that the donor atoms bind tightly to the metal hindering cleavage of but one of the bonds at the palladium metal center, in a similar fashion to the thiosemicarbazone palladacycles,^[29]^ keeping the metal away from the media, and disabling the performance of the compound as a pre‐catalyst. The results showed that this is indeed the case, but for two of the complexes tested, albeit under harsher conditions. The results showed that the bidentate ligands were more active as catalysts than the tridentate analogues.

Herein, we describe the preparation and characterization of single and dinuclear palladacycles with chloride or bromide substituents on the metallated ring some of which have been successfully tested as catalysts for the Suzuki‐Miyaura cross‐coupling reaction using a mixture of water/organic solvent. The compounds were characterized by microanalytical data (C, H, N), and by IR and 1H‐NMR, ^31^P‐{^1^H} NMR spectroscopies. Three compounds, **4 h**, **1 c** and **1 e**, were also studied by X‐Ray diffraction analysis.

## Results and Discussion

**Scheme 1:**
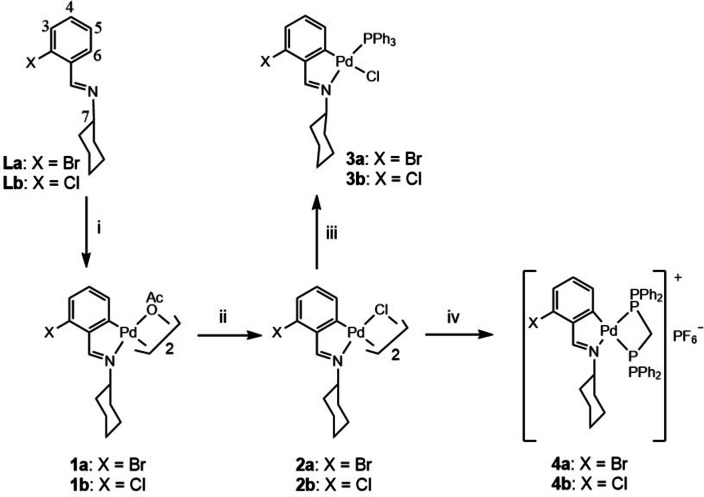
‐ i) Pd(OAc)_2_, acetic acid, 65° C; ii) NaCla_(aq)_, aetone; iii) PPh_3_, acetone; iv) dppm, ammonium hexafluorophosphate, acetone.

**Scheme 2:**
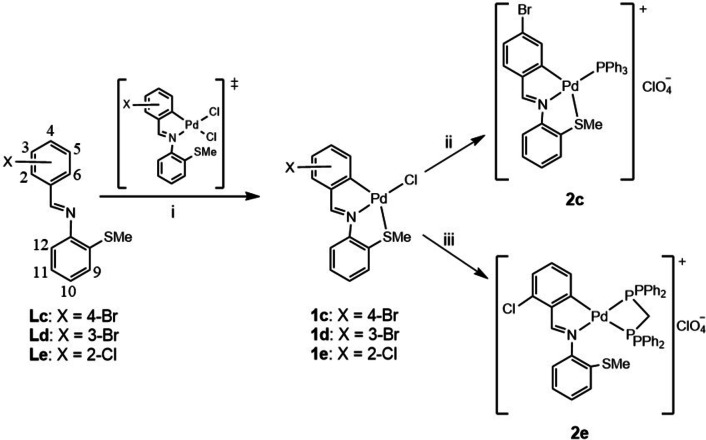
‐ i) Li_2_[PdCl_4_], methanol, reflux; ii) PPh_3_, silver perchlorate, acetone, iii) dppm, silver perchlorate, acetone.

For the convenience of the reader, the compounds and reactions are shown in Schemes 1 and 2. The compounds were characterized by elemental analysis (C, H, N, S) and by IR and by ^1^H and ^31^P‐{^1^H} NMR spectroscopy. (See Experimental). The ligands **La**‐**Le** were synthesized by refluxing the corresponding aldehyde and amine in chloroform in a modification of a Dean‐Stark apparatus. Treatment of the ligands **La**‐**Lb** with palladium(II) acetate in acetic acid at 65 °C for 8 h gave after work up the μ‐acetate dinuclear complexes **La‐Lb**. The palladium atom was coordinated to the imine double bond through the nitrogen lone pair as shown by the shift of the υ(C=N) stretching vibration to lower frequency in the IR spectra^[30]^ as compared to the free ligand spectra, and an upfield shift of the HC=N resonance in the ^1^H NMR spectra.^[31]^ The υ_as_(COO) and υ_s_(COO) values were consistent with bridging acetate groups;^[32]^ the latter were arranged in a *trans* geometry as two equivalent moieties as evidenced by a singlet resonance in the ^1^H NMR spectra. Furthermore, the H6 resonance was absent consequent on metalation of the C6 carbon atom. Reaction of palladacycles **1 a**, **1 b** with sodium chloride gave compounds **2 a**, **2 b**. The spectroscopic results were in accordance with removal of the μ‐acetate ligands. Treatment of the halide‐bridged complexes with PPh_3_ or with Ph_2_PCH_2_PPh_2_ (dppm) in a palladacycle/phosphine 1 : 2 ratio and silver perchlorate yielded the phosphine derivatives **3 a**, **3 b** and **4 a**, **4 b**, respectively. The ^31^P‐{^1^H} NMR spectra showed a singlet resonance (**3 a**, **3 b**), on the one hand, and two doublets (**4 a**, **4 b**) for the two inequivalent phosphorus nuclei, on the other; in the latter case the higher frequency doublet to the phosphorus nucleus trans to the imine nitrogen, based on the assumption that a ligand of greater trans influence shifts the ^31^P resonance in *trans* to lower frequency.^[33]^

Treatment of the ligands **Lc**‐**Le** with lithium tetrachloropalladate gave the palladacycles **1 c**‐**1 e** as air‐stable solids, with the ligands bonded to the metal center in a tridentate [C,N,S] fashion. The IR spectra showed the υ(C=N) and υ(Pd−Cl) stretches ca. 1580 and 310 cm^−1^, respectively, which together with the upfield shift of the C5(H) resonance in the ^1^H NMR supported nitrogen coordination to the metal center, *vide supra*. In the case of 1c metallation of the ligand was confirmed from the absence of the AA'XX' system of the *para*‐substituted phenyl ring, and the three remaining proton resonances were unequivocally assigned. The S*Me* resonance was shifted downfield in the ^1^H NMR spectra, in agreement with Pd−S coordination. Reaction of complex **1 c** with triphenylphosphine or of **1 e** with Ph_2_PCH_2_PPh_2_ did not show cleavage of the Pd−S nor the Pd−Cl bonds, even with a large excess of the corresponding phosphine ligand. The phosphine could be coordinated to the metal atom only after abstraction of the chlorine ligand, which was produced by treatment of compound **1 c** or **1 e** with silver perchlorate, and subsequent addition of the phosphine ligand to give **2 c** and **2 e**, the latter as a 1 : 1 electrolyte. The removal of the chlorine ligand was confirmed by the absence of the υ(Pd−Cl) band. The H(5) resonance in the ^1^H NMR spectrum showed an upfield shift and appeared as a multiplet, due to coupling to the ^31^P nucleus; the ^31^P resonances were assigned accordingly, *vide supra*.

## Crystal structure of 4 a

**Figure 1:**
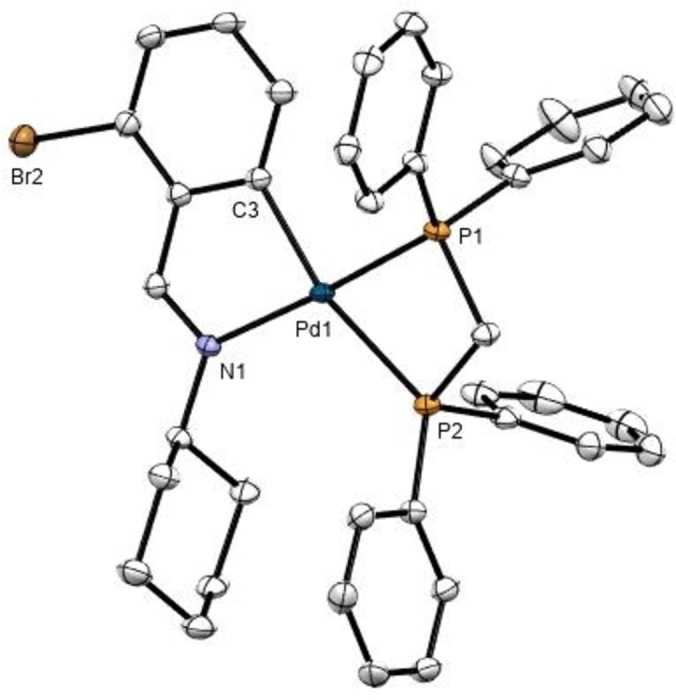
ORTEP drawing of compound **4 a** with thermal ellipsoid plot shown at 50 % probability level. Hydrogen atoms, solvent molecules and counterions have been omitted for clarity. Selected bond distances (Å) and angles (°): Pd(1)‐C(3) 2.033(3), Pd(1)‐N(1) 2.120(2), Pd(1)‐P(1) 2.2452(8), Pd(1)‐P(2) 2.3827(8), P(2)‐Pd(1)‐P(1) 72.12(3), C(3)‐Pd(1)‐P(1) 96.17(9), C(3)‐Pd(1)‐N(1) 80.9711), N(1)‐Pd(1)‐P(2) 111.02(7), N(1)‐Pd(1)‐P(1) 176.10(7), C(3)‐Pd(1)‐P(2) 166.53.

Suitable crystals were grown by slow evaporation of a dichloromethane/n‐hexane solution. The asymmetric unit for the crystal structure of **4 a** (Figure 1 and Table 1) consists of one molecule and one hexafluorophosphate ion with the palladium (II) atom, bonded in a square‐planar environment to two chelating ligands: bidentate [C,N] Schiff base ligand and a Ph_2_PCH_2_PPh_2_‐*P*,*P* diphosphine. The sum of angles at the metal center is *ca*. 360°, with the most significant distortion in the somewhat reduced “bite” angle C(1)‐Pd(1)‐N(1), 80.97(11)° upon chelation. The P(1)–Pd(1)–P(2) bond angle 72.12(3)° reflects the strain of the four‐membered chelate ring of the phosphine. The Pd(1)‐N(1) bond length, *ca*. 2.120(2) Å is longer than the single bond expected value of 2.011 Å, due to the *trans* influence of the phosphine ligand. The Pd(1)‐C(1) bond length of 2.033(3) Å, shorter than the expected value of 2.081 Å^[34]^ cita justifying some degree of partial multiple‐bond on the Pd–C bond.^[35]^ Intermolecular interactions were observed between the bromine atoms and the phosphine phenyl ring of a neighboring molecule (Table 2).

**Table 1:** Crystallographic data for **4 a**, **1 c** and **1 e**.

Compound	4 a	1 c	1 e
Empirical formula	C_38_H_37_BrF_6_NP_3_Pd	C_56_H_44_Br_4_Cl_4_N_4_Pd_4_S_4_	C_28_H_22_Cl_4_N_2_Pd_2_S_2_
Formula weight	900.955	1788.350	805.273
Temperature/K	100.00	100.00	100.00
Crystal system	monoclinic	trigonal	monoclinic
Space group	P2_1_/c	R‐3	P2_1_/c
a/Å	12.3331(7)	28.1329(17)	14.3485(3)
b/Å	13.8140(8)	28.1329(17)	18.2981(3)
c/Å	22.5392(14)	9.5511(7)	10.9824(2)
α/°	90	90	90
β/°	104.621(2)	90	99.9459(10)
γ/°	90	120	90
Volume/Å^3^	3715.6(4)	6546.6(7)	2840.10(9)
Z	4	4.5	4
ρ_calc_g/cm^3^	1.611	2.041	1.883
μ/mm^‐1^	1.763	4.333	15.236
F(000)	1805.6	3872.9	1598.1
Crystal size/mm^3^	0.13×0.06×0.04	0.22×0.04×0.04	0.1×0.05×0.03
Radiation	Mo Kα (λ=0.71073)	Mo Kα (λ=0.71073)	Cu Kα (λ=1.54178)
2Θ range for data collection/°	4.38 to 58.26	4.58 to 56.56	6.26 to 149
Index ranges	‐16≤h≤16, −18≤k≤18, −30≤l≤30	‐37≤h≤37, −37≤k≤37, −12≤l≤12	‐17≤h≤17, −22≤k≤22, −13≤l≤13
Reflections collected	213238	55099	81641
Independent reflections	10000 [R_int_=0.0928, R_sigma_=0.0314]	3633 [Rint=0.0815, Rsigma=0.0333]	5797 [R_int_=0.0922, R_sigma_=0.0354]
Data/restraints/parameters	10000/0/451	3633/0/173	5797/0/345
Goodness‐of‐fit on F^2^	1.060	1.049	1.002
Final R indexes [I>=2σ (I)]	R_1_=0.0381, wR_2_=0.0862	R1=0.0392, wR2=0.0862	R_1_=0.0325, wR_2_=0.0726
Final R indexes [all data]	R_1_=0.0551, wR_2_=0.0966	R1=0.0559, wR2=0.0930	R_1_=0.0464, wR_2_=0.0788
Largest diff. peak/hole / e Å^−3^	0.92/‐1.72	2.24/‐1.74	0.74/‐1.05

**Table 2:** C−Br interactions (Å) of compound **4 a**.

**Y−X−Cg**	**Y−X**	**X−Cg**	**Y−Cg**	**<(Y−X−Cg)°**
**C5−Br1−Cg1**	1.884	3.316	5.173	167.86

Symmetry operation −1+x, y, z.

## Crystal structures of 1 c and 1 e

**Figure 2:**
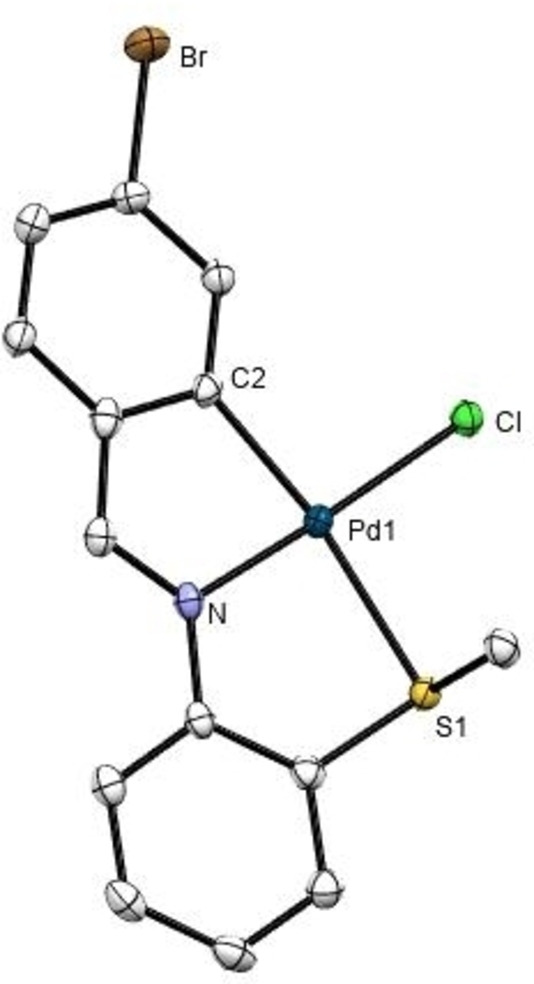
ORTEP drawing of compound **1 c** with thermal ellipsoid plot shown at 50 % probability level. Hydrogen atoms and solvent molecules have been omitted for clarity. Selected bond distances (Å) and angles (°): Pd(1)‐C(2) 1.992(4), Pd(1)‐N 2.013(4), Pd(1)‐S(1) 2.3802(11), Pd(1)‐Cl 2.3129(11), S(1)‐Pd(1)‐Cl 96.99(4), N−Pd(1)‐S(1) 85.03(11), N−Pd(1)‐Cl 175.70(11), C(2)‐Pd(1)‐S(1) 166.30(13), C(2)‐Pd(1)‐Cl 95.81(13), C(2)‐Pd(1)‐N 81.74(17).

**Figure 3:**
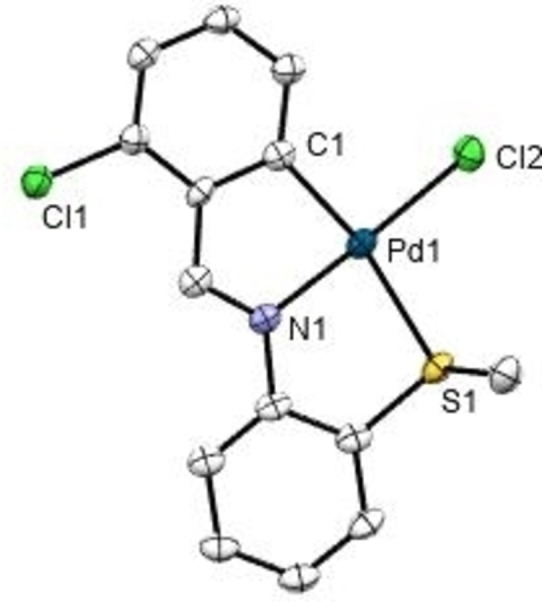
ORTEP drawing of compound **1 e** with thermal ellipsoid plot shown at 50 % probability level. Hydrogen atoms and solvent molecules have been omitted for clarity. Selected bond distances (Å) and angles (°): Pd(1)‐C(1) 1.992(4), Pd(1)‐N(1) 2.001(4), Pd(1)‐S(1) 2.3676(11), Pd(1)‐Cl(2) 2.2998(12), S(1)‐Pd(1)‐Cl(2) 96.11(4), N(1)‐Pd(1)‐S(1) 85.49(11), N(1)‐Pd(1)‐Cl(2) 177.29(10), C(1)‐Pd(1)‐S(1) 167.59(13), C(1)‐Pd(1)‐Cl(2) 95.18(13), C(2)‐Pd(1)‐N(1) 82.30(16).

The crystal structures of **1 c** and **1 e** (Figures 2 and 3, respectively, and Table 1) comprise discrete molecules separated by van der Waals distances. The asymmetric unit for **1 e** contains two unique molecules. The palladium(II) atom is bonded in a square‐planar environment to four different donors, three of which pertain to the tridentate imine ligand: the aryl C(6) carbon, the imine N(1) nitrogen, and the S(1) sulfur; and the fourth is the chlorine atom Cl(1). The structures show four fused rings, two of which share the metal center. The two five‐membered organometallic and coordination rings share the Pd(1)−N(1) bond; whilst each in turn have a carbon‐carbon bond in common with the two phenyl rings. All bond lengths are within the expected range, with allowance for the somewhat shorter Pd(1)‐C(1) length, of 2.044(4) Å, *vide supra*. The angles between adjacent atoms in the palladium coordination sphere are *ca*. 90° with the most noticeable distortion in the N(1)Pd(1)C(6) angle of 81.7(2)° and 81.7(2)°, due to chelation; the sum of angles around the palladium atom is 359.57° (**1 c**) and 359.08° (**1 e**).

## Catalysis

To study the catalytic activity of the new imine palladacycles they were probed as possible pre‐catalysts in the Suzuki‐Miyaura coupling (SMC) to give the resulting biaryl species. All were tested for a standard coupling reaction in order to stablish the most efficient catalysts. Thus, treatment of 4‐bromoacetophenone with phenylboronic acid in THF:water (2 : 1) or EtOH:water (2 : 1) at 80–100 °C for 24–36 h in the presence of 4 % mol catalyst (expressed in percent of Pd) and base, K_2_CO_3_, gave the biphenyl coupled product 4‐phenylacetophenone in 100–60 % (Table 3); conversions were calculated using the integral of the −CO*Me* resonance. Those that showed acceptable activity are included in Table 3.

**Table 3:** Catalytic activity of the compounds.^a^

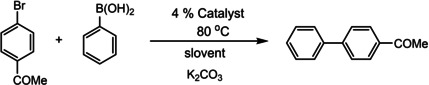					
Entry	[cat]	T(°C)	Time (h)	solvent	Conv. (%)^b^
**1**	Pd(OAc)_2_	80	24	THF/H_2_O	64 %
**2**	1 a	80	24	THF/H_2_O	100 %
**3**	1 b	80	24	THF/H_2_O	100 %
**4**	2 a	80	24	THF/H_2_O	95 %
**5**	2 b	80	24	THF/H_2_O	94 %
**6**	3 a	80	24	THF/H_2_O	12 %
**7**	3 b	80	24	THF/H_2_O	9 %
**8**	4 a	80	17	EtOH/H_2_O	100 %
**9**	4 b	80	17	EtOH/H_2_O	100 %
**10**	1 c	100	36	EtOH/H_2_O	70 %
**11**	1 e	100	36	EtOH/H_2_O	65 %

^a^ Reaction conditions: 1 mmol aryl halide, 1.2 eq. phenylboronic acid, 2 eq. base K_2_CO_3_, 2 % mol palladacycle; 2 cm^3^ solvent. ^**b**^determined by NMR.

Increasing the water percentage of the solvent mixture ratio produced less satisfactory results and as the proportion of water increased the efficiency of the catalysis weakened. Nevertheless, that water may be used as a component of the solvent system opens the hope for a more environmentally friendly reaction medium. The dinuclear acetate‐bridged complexes **1 a**, **1 b** (entries 2, 3) showed good catalytic activity and with slightly better yields than the analogous dinuclear halide‐bridged species **2 a**, **2 b** (entries 4, 5). Rather surprisingly, compounds with triphenylphosphine **3 a**, **3 b** (entries 6, 7) did not give suitable conversions, <15 %, and were not considered further. However, complexes bearing the diphosphine Ph_2_PCH_2_PPh_2_ (dppm) **4 a**, **4 b** (entries 8, 9) produced the best results with yields of 100 % at 80 °C, but bringing the reaction time down to only 17 h. The compounds with tridentate [C,N,S] imine ligands **1 c**, **1 e** (entries 10, 11) showed a lower activity than their bidentate [C,N] counterparts; despite reaching a conversion of *ca*. 70 % this was produced at higher temperature and over a longer reaction time. In any case, the conversion percentages, excepting **3 a** and **3 b**, were better the for palladium(II) acetate as catalyst for the present cross‐coupling reaction under analogous conditions (entry 1). The results show lower conversions than those found for all the compounds tested herein inclusive of the tridentate ligand palladacycles.

## Experimental

### General Procedures

Solvents were used without previous purification. Chemicals were reagent grade. The diphosphine Ph_2_PCH_2_PPh_2_ (dppm) was purchased from Sigma−Aldrich. Elemental analyses were carried out on a THERMO FINNIGAN, model FLASH 1112. IR spectra were recorded with a JASCO FT/IR−4600 spectrometer equipped with an ATR, model ATR−PRO ONE. ^1^H NMR spectra in solution were recorded in CDCl_3_ or Acetone‐d_6_ at room temperature on Varian Inova 400 spectrometer operating at 300.14 MHz. ^31^P‐{^1^H} NMR spectra were recorded at 202.46 MHz on a Bruker AMX 500 spectrometer. All chemical shifts are reported downfield from standards using the solvent signal (CDCl_3_, δ ^1^H=7.26 ppm, dmso‐d_6_ δ^1^H=2.50, and acetone‐d_6_ δ^1^ H=2.05) as reference and for ^31^P relative to external H_3_PO_4_ (85 %). All the NMR experiments were done using 5 mm o.d. tubes.

### Preparation of the Ligands

The corresponding aldehyde and cyclohexylamine or 2‐thiomethyl aniline as appropriate were added together in chloroform (*ca*. 40 cm^3^) in a 100 mL round‐bottomed flask. The resulting mixture was stirred under reflux for 4 h, after which the solvent was reduced to low volume and the precipitate formed was filtered off and dried under vacuum.

**La**. Yield 87 % Anal. Found: C: 58.5; H: 6.0; N: 5.3 %, C_13_H_16_NBr (266.18 g/mol) requires C: 58.7; H: 6.1; N: 5.3 %. IR: υ(C=N) 1625 m cm^−1^. ^1^H NMR (400 MHz, CDCl_3_) δ(ppm): 8.68 (s, 1H, *H*C=N), 8.03 (dd, ^3^ *J*(H6H5)=7.8 Hz, ^4^ *J*(H6H4)=2.3 Hz, 1H, H6), 7.56 (d, ^3^ *J*(H3H4)=7.8 Hz, 1H, H3), 7.33 (t, ^3^ *J*(H5H6)=7.8 Hz, 1H, H5), 7.24 (t, ^3^ *J*(H4H3)=7.8 Hz, 1H, H4), 3.31 (m, 3 *J*(HH)=10.4 Hz, 1H, N‐C*H7*), 1.94–1.20 (m, 10H, Cy).

**Lb**. Yield 91 % Anal. Found: C: 70.4; H: 7.1; N: 6.1 %, C_13_H_16_NCl (221.23 g/mol) requires C: 70.4; H: 7.3; N: 6.2 %. IR: υ(C=N) 1620 m cm^−1^. ^1^H NMR (400 MHz, CDCl_3_) δ(ppm): 8.68 (s, 1H, *H*C=N), 8.03 (dd, ^3^ *J*(H6H5)=7.8 Hz, ^4^ *J*(H6H4)=2.3 Hz, 1H, H6), 7.56 (d, ^3^ *J*(H3H4)=7.8 Hz, 1H, H3), 7.33 (t, ^3^ *J*(H5H6)=7.8 Hz, 1H, H5), 7.24 (t, 3 *J*(H4H3)=7.8 Hz, 1H, H4), 3.31 (m, ^3^ *J*(HH)=10.4 Hz, 1H, N‐C*H7*), 1.94–1.20 (m, 10H, Cy).

**Lc**. Yield 75 % Anal. Found: C: 54.7; H: 4.0; N: 4.5; S: 10.5 %, C_14_H_12_NBrS (306.22 g/mol) requires C: 54.9; H: 4.0; N: 4.6; S: 10.5 %. IR: υ(C=N) 1625 m cm^−1^. ^1^H NMR (400 MHz, dmso‐d_6_) δ(ppm): 8.57 (s, 1H, HC=N), 7.89 (d, *N*=8.5 Hz, 2H, H2, H6), 7.75 (d, *N*=8.5 Hz, 2H, H3, H5), 7.24 (m, 4H, H9, H10, H11, H12), 2.4 (s, 3H, SMe)

**Ld**. Yield 85 % Anal. Found: C: 54.8; H: 4.0; N: 4.4; S: 10.5 %, C_14_H_12_NBrS (306.22 g/mol) requires C: 54.9; H: 4.0; N: 4.6; S: 10.5 %. IR: υ(C=N) 1628 m cm^−1^. ^1^H NMR (400 MHz, dmso‐d_6_) δ(ppm): δ 8.58 (s, 1H, HC=N), 8.13 (s, 1H, H2), 7.95 (d,^3^JH6H5=8.0 Hz, 1H, H6), 7.75 (d, ^3^JH4H5=8.0 Hz, 1H, H4), 7.50 (m, 1H, H5), 7.27 (m, 4H, H9, H10, H11, H12), 2.42 (s, 3H, SMe).

**Le**. Yield 95 % Anal. Found: C: 64.0; H: 4.5; N: 5.2; S: 12.3 %, C_14_H_12_NClS (261.77 g/mol) requires C: 64.2; H: 4.6; N: 5.4; S: 12.3 %. IR: υ(C=N) 1623 m cm^−1^. ^1^H NMR (400 MHz, dmso‐d_6_) δ(ppm): δ 8.81 (s, 1H, HC=N), 8.19 (dd, ^3^JH6H5=7.8 Hz), ^4^J(H6H4=1.8 Hz, 1H, H6), 7.59 (dd, ^3^JH3H4=7.8 Hz, ^4^JH3H5=1.8 Hz, 1H, H3), 7.53 (m, 1H, H4), 7.38 (m, 1H, H5), 7.29 (m, 4H, H9, H10, H11, H12), 2.41 (s,3H, SMe).

### Preparation of the Complexes

**Synthesis of 1 a**. In round‐bottomed flask palladium(II) acetate 100 mg (0.44 mmol) and ligand La 110 mg (100 mmol) were added together in toluene (40 cm3), and the resulting solution was heated to 65 °C for 3 h. After cooling to room temperature, the solution was filtered to remove any black palladium formed and the solvent removed under vacuum. The resulting product was recrystallized from dichloromethane/n‐hexane, washed with cold ethanol, and dried under vacuum. Yield 95 % Anal. Found: C: 41.6; H: 4.0; N: 3.1 %, C_30_H_36_N_2_Br_2_O_4_Pd_2_ (861.26 g/mol) requires C: 41.8; H: 4.1; N: 3.3 %. IR: υ(C=N) 1580 cm^−1^, υ_as_(COO) 1557 cm^−1^, υ_s_(COO) 1402 cm^−1^. ^1^H NMR (400 MHz, CDCl3) δ 7.71 (s, 2H, Hi), 7.12 (d, ^3^J(H3H4)=7.7 Hz, 2H, H3), 7.01 (d, ^3^J(H5H4)=7.7 Hz, 2H, H5), 6.86 (t, ^3^J(H4H3)=7.7 Hz, 2H, H4), 2.96 (m, 2H, N‐CH−Cy), 2.14 (s, 6H, OAc), 0.8–2.45(m, 20H, Cy).

Compound **1 b** was prepared in a similar fashion.

**1 b**. Yield 95 % Anal. Found: C: 46.5; H: 4.6; N: 3.4 %, C_30_H_36_N_2_Cl_2_O_4_Pd_2_ (772.37 g/mol) requires C: 46.7; H: 4.7; N: 3.6 %. IR: υ(C=N) 1577 cm^−1^, υ_as_(COO) 1558 cm^−1^, υ_s_(COO) 1406 cm^−1^. ^1^H NMR (400 MHz, CDCl3) δ 7.74 (s, 2H, HC=N), 6.96 (s, 6H, H3, H4, H5), 2.99 (t, 3 J(HH)=12.0 Hz, 2H, N‐CH−Cy), 2.14 (s, 6H, OAc), 0.8–2.50 (m, 20H, Cy).

**Synthesis of 2 a**. An aqueous solution of sodium chloride (25 cm^3^, *ca*. 0.05 M) was added dropwise to an acetone solution of complex 1a (15 cm^3^). The mixture was stirred for 2 h, and a yellow solid precipitated, which was filtered off, washed with cold water and dried under vacuum. Yield 95 % Anal. Found: C: 38.3; H: 3.5; N: 3.4 %, C_26_H_30_N_2_Br_2_Cl_2_Pd_2_ (814.09 g/mol) requires C: 38.4; H: 3.7; N: 3.4 %. IR: υ(C=N) 1606 cm^−1^, υ(Pd‐Cl_transN_) 340 cm^−1^, υ(Pd‐Cl_transC_) 270 cm^−1^. ^1^H NMR (400 MHz, CDCl_3_) δ 8.20 (s, 2H, HC=N), 7.34 (d, ^3^ *J*(H3H4)=7.9 Hz, 2H, H3), 7.17 (d, ^3^ *J*(H5H4)=7.8 Hz, 2H, H5), 6.86 (t, ^3^ *J*(H4H3)=7.9 Hz, 2H, H4), 3.71 (m, 2H, N‐CH−Cy). 1.28–2.25(m, 20H, Cy).

Compound **2 b** was prepared analogously.

**2 b**. Yield 95 % Anal. Found: C: 42.9; H: 4.1; N: 3.7 %, C_26_H_30_N_2_Cl_4_Pd_2_ (725.18 g/mol) requires C: 43.1; H: 4.2; N: 3.9 %. %. IR: υ(C=N) 1609 cm^−1^, υ(Pd‐Cl_transN_) 340 cm^−1^, υ(Pd‐Cl_transC_) 274 cm^−1^. ^1^H NMR (400 MHz, CDCl_3_) δ 8.21 (s, 2H, HC=N), 6.97 (s, 3H, H3, H4, H5), 3.71 (m, 2H, N‐CH−Cy), 1.0–2.25 (m, 20H, Cy).

**Synthesis of 3 a**. Complex **2 a** (25 mg, 34 mmol) and triphenylphosphine (16 mg, 61 mmol) were added in acetone (10 cm^3^), and the resulting mixture was stirred at room temperature for 3 h. After reducing to low volume, the resulting solid was recrystallized from dichloromethane/n‐hexane. Yield 95 % Anal. Found: C: 55.2; H: 4.4; N: 2.1 %, C_31_H_30_NBrClPPd (669.33 g/mol) requires C: 55.6; H: 4.5; N: 2.1 %. IR: υ(C=N) 1674 cm^−1^, υ(Pd−Cl) 306 cm^−1^. ^1^H NMR (400 MHz, CDCl_3_) δ 8.62 (d, ^4^ *J*(PH)=8.4 Hz, 1H, HC=N), 7.76 (d, ^3^ *J*(HH)=7.5 Hz, 6H, PPh_3_), 7.45 (d, ^3^ *J*(HH)=7.5 Hz, 3H, PPh_3_), 7.39 (d, ^3^ *J*(HH)=7.5 Hz, 6H, PPh_3_), 7.01 (m, 1H, H4), 6.34 (d, N=2.5 Hz, 2H, H3 and H5), 4.52 (m, ^3^ *J*(HH)=12.3 Hz, 1H, N‐CH−Cy), 0.8−2.30 (m, 10H, Cy). ^31^P NMR (δ ppm, CDCl_3_) δ 42.58.

Compound **3 b** was prepared analogously.

**3 b**. Yield 95 % Anal. Found: C: 59.6; H: 4.7; N: 2.2 %, C_31_H_30_NCl_2_PPd (624.88 g/mol) requires C: 59.6; H: 4.8; N: 2.2 %. IR: υ(C=N) 1574 cm^−1^, υ(Pd−Cl) 300 cm^−1^. ^1^H NMR (400 MHz, CDCl_3_) δ 8.62 (d, ^4^ *J*(PH)=5.2 Hz, 1H, HC=N), 7.73 (d, ^3^ *J*(HH)=7.6 Hz, 6H, PPh_3_), 7.53–7.39 (m, 9H, PPh_3_), 6.84 (d, ^3^ *J*(H3H4)=7.9 Hz, 1H, H3), 6.44 (t, ^3^ *J*(H4H3)=^3^ *J*(H4H5)=7.9 Hz, 1H, H4), 6.30 (d, ^3^ *J*(H5H4)=7.9 Hz, 1H, H5), 4.53 (m, ^3^ *J*(HH)=11.6 Hz, 1H, N−CH−Cy), 0.9–2.30 (m, 10H, Cy). ^31^P NMR (δ ppm, CDCl_3_) δ 42.80.

**Synthesis of 4 a**. Complex **2 a** (30 mg, 36 mmol), Ph_2_PCH_2_PPh_2_ (dppm) (28 mg, 73 mmol) and NH_4_PF_6_ (12.0 mg, 73 mmol) were added in acetone (10 cm^3^). The resulting mixture was stirred at room temperature for 3 h. After reducing to low volume, the resulting solid was recrystallized from dichloromethane/n‐hexane. Yield 95 % Anal. Found: C: 50.5; H: 4.0; N: 1.4 %, C_38_H_37_NBrF_6_P_3_Pd (900.96 g/mol) requires C: 50.7; H: 4.1; N: 1.6 %. IR: υ(C=N) 1560 m cm^−1^. ^1^H NMR (400 MHz, CDCl_3_) δ 8.67 (d, ^4^ *J*(PH)= 5.5 Hz, 1H, HC=N), 7.83–7.34 (m, 20H, PPh_2_), 7.22 (d, ^3^ *J*(H3H4)=7.5 Hz, 1H, H3), 6.72 (t, ^3^ *J*(H4H3)=^3^ *J*(H4H5)=7.5 Hz 1H, H4), 6.64 (d, ^3^ *J*(H5H4)=7.5 Hz, 1H, H5), 4.30 (t, ^2^ *J*(HP)=9.9 Hz, 2H, PCH_2_P), 3.38 (m, *J*=12.5 Hz, 1H, N‐CH−Cy), 0.6–2.20 (m, 10H, Cy).

^31^P NMR (δ ppm, CDCl_3_) δ −4.82 (d, *J* = 61.0 Hz), −29.22 (d, *J* = 61.0 Hz), −141.28 (heptuplet, PF_6_^−^).

Compound **4 b** was prepared analogously.

**4 b**. Yield 95 % Anal. Found: C: 53.2; H: 4.2; N: 1.6 %, C_38_H_37_NClF_6_P_3_Pd (856.50 g/mol) requires C: 53.3; H: 4.4; N: 1.6 %. IR: υ(C=N) 1562 m cm^−1^. ^1^H NMR (400 MHz, CDCl_3_) δ 8.67 (br s, 1H, HC=N), 7.82–7.33 (m, 20H, PPh_2_), 7.03 (d, ^3^ *J*(H3H4)=7.8 Hz, 1H, H3), 6.81 (t, ^3^ *J*(H4H5)=^3^ *J*(H4H3)=7.8 Hz, 1H, H4), 6.59 (d, ^3^ *J*(H5H4)=7.8 Hz, 1H, H5), 4.29 (br s, 2H, PCH_2_P), 3.41 (m, 1H, N−CH−Cy), 0.6–2.37 (m, 10H, Cy). ^31^P NMR (δ ppm, CDCl_3_) δ −3.50 (d, *J*=69.7 Hz), −28.96 (d, *J*=69.7 Hz), −143.48 (heptuplet, PF_6_^−^).

**Synthesis of 1 c**. Palladium chloride and lithium chloride were added together in oxygen‐free methanol in a carousel flask under argon, and the mixture was stirred at room temperature until a reddish color appeared. Then, ligand **Lc** (104 mg (0.40 mmol) was added, and the reaction mixture stirred under refluxed for 4 h. After cooling to room temperature, and one equivalent of sodium acetate was added, which produced the instantaneous formation of an orange solid that was filtered off and dried under vacuum. Yield 95 % Anal. Found: C: 37.4; H: 2.5; N: 3.2; S; 7.1 %, C_14_H_11_NBrClSPd (447.09 g/mol) requires C: 37.6; H: 2.5; N: 3.3; S; 7.2 %. IR: υ(C=N) 1550, υ(Pd−Cl) 315 cm^−1^. ^1^H NMR (400 MHz, dmso‐d_6_) δ 9.35 (s, 1H, HC=N), 8.12 (d, ^3^J(H2H3)=7.7 Hz, 1H, H2), 7.94 (d, ^3^J(H3H2)=7.7 Hz, 1H, H3), 7.89 (d, ^3^J=7.9 Hz, 1H), 7.75 (s, 1H, H5), 7.67 (t, ^3^J(H10H11)=^3^J(H10H9)=8.0 Hz, 1H, H10), 7.59 (d, ^3^J(H9H10)=8.0 Hz, 1H, H9) 7.54 (t, ^3^J(H11H10)=^3^J(H11H12)=8.0 Hz, 1H, H11), 7.49 (d, ^3^J(H12H11)=8.0 Hz, 1H, H12) 7.39 (d, ^3^J=8.0 Hz, 1H), 2.89 (s, 3H, SMe).

Complexes **1 d** and **1 e** were prepared similarly.

**1 d**. Found: requires C: 37.5; H: 2.4; N: 3.3; S; 7.1 %, C_14_H_11_NBrClSPd (447.09 g/mol) requires C: 37.6; H: 2.5; N: 3.3; S; 7.2 %. IR: IR: υ(C=N) 1565, υ(Pd−Cl) 310 cm^−1^. ^1^H NMR (400 MHz, acetone‐d_6_) δ 9.23 (s, 1H, HC=N), 8.06 (d, ^3^ *J* (H4H5)=8.1 Hz, 1H, H4), 7.85 (d, ^3^ *J* (H5H4)=8.1 Hz, 1H, H5), 7.74 (s, 1H, H2), 7.68 (m, 1H, H10), 7.50 (d, ^3^ *J* (H9H10)=7.9 Hz, 1H, H9), 7.40 (m, 1H, H11), 7.33 (d, ^3^ *J* (H12H11)=7.9 Hz, H12), 7.08 (d, ^3^ *J*=8.1 Hz, 1H). 2.87 (s, 3H, SMe).

**1 e**. Yield 95 % Anal. Found: C: 41.7; H: 2.6; N: 3.4; S: 7.8 %, C_14_H_11_NCl_2_SPd (402.63 g/mol) requires C: 41.8; H: 2.8; N: 3.5; S: 8.0 %. IR: υ(C=N) 1625 m cm^−1^. ^1^H NMR (400 MHz, acetone‐d_6_) δ 9.30 (s, 1H, HC=N), 8.22 (d, ^3^ *J*H3H4 = 8.0 Hz, 1H, H3), 7.85 (d, ^3^ *J* (H5H4)=8.0 Hz, 1H, H5), 7.69 (d, ^3^ *J* (H9H10)=7.6 Hz, 1H, H9), 7.60 (t, ^3^ *J* (H4H5)=^3^ *J* (H4H3)=8.0 Hz, 1H, H4), 7.56 (m, 1H, H10), 7.20 (t, ^3^ *J* (H11H12)=^3^ *J* (H11H10)=7.7 Hz, 1H, H11), 7.11 (d, ^3^ *J* (H12H11)=7.7 Hz, 1H, H12), 2.80 (s, 3H, SMe).

**Synthesis of 2 c**. In a round‐bottomed flask complex **1 c** (10 mg, 20 mmol) and silver perchlorate (4 mg, 20 mmol) were in acetone (10 cm^3^) and the mixture was stirred for 4 h. Then, after removal of the silver chloride formed triphenylphpohine (5 mg, 20 mmol) and stirring continued for 24 h. The residue was recrystallized from dichloromethane/n‐1hexane and dried under vacuum. Yield 60 % Anal. Found: C: 49.6; H: 3.4; N: 1.8; S: 4.1 %, C_32_H_26_NBrClO_4_PSPd (773.37 g/mol) requires C: 49.7; H: 3.4; N: 1.8; S: 4.2 %. IR: υ(C=N) 1570 cm^−1^. ^1^H NMR (400 MHz, acetone‐d_6_) δ 9.48 (s, 1H, HC=N), 8.22 (d, ^3^ *J*H3H4 = 8.0 Hz, 1H, H3), 6.57 (d, ^4^ *J*PH5=4.9 Hz, 1H, H5), 8.16–7.54 (m, Ph), 7.45 (m, 1H, H2), 7.37 (m, 1H, H3), (s, 3H, SMe). ^31^P NMR (δ ppm, CDCl_3_) δ −4.82 (d, *J*=61.0 Hz).

**Synthesis of 2 e**. Complex **1 e** (20 mg, 49 mmol) and silver perchlorate (10 mg, 49 mmol) were in acetone (10 cm^3^) and the mixture was stirred for 4 h. Then, after removal of the silver chloride formed Ph_2_PCH_2_PPh_2_ (dppm) (18 mg, 49 mmol) and stirring continued for 24 h. The residue was recrystallized from dichloromethane/n‐hexane and dried under vacuum. Yield 50 % Anal. Found: C: 54.9; H: 3.9; N: 1.7; S: 3.9 %, C_39_H_33_NCl_2_O_4_P_2_SPd requires (851.02 g/mol) C: 55.0; H: 3.9; N: 1.7; S: 3.8 %. IR: υ(C=N) 1574 cm^−1^. ^1^H NMR (400 MHz, acetone‐d_6_) δ 9.08 (s, 1H, HC=N), 8.0‐6.82 (m, Ph), 4.40 (br, m, 2H, PCH_2_P), 2.80 (s, 3H, SMe). ^31^P NMR (δ ppm, CDCl_3_) δ 51.44 (d, *J*=69.7 Hz), 24 (d, *J*=25 Hz),

## Conclusions

Herein, we have shown that reaction of halide substituted bidentate [C,N] imine ligands with palladium(II) acetate produces double nuclear Schiff base palladacycles with bridging acetate ligands, [Pd(C_sp2_,N‐imine)(*μ*‐OAc)]_2_, from them the corresponding *μ*‐chloride analogues, [Pd(C_sp2_,N‐imine)(*μ*‐Cl)]_2_, are readily prepared. Reaction of the latter with tertiary mono‐ or bidentate phosphines, PPh_3_P and Ph_2_PCH_2_PPh_2_ (dppm), respectively, produces the single nuclear complexes [PdCl(C_sp2_,N‐imine)(PPh_3_)] and [Pd(C_sp2_,N‐imine)(dppm‐*P*,*P*)(PF_6_)]. The molecular structure of **4 a** has been studied by single‐crystal X‐ray diffraction. Furthermore, treatment of the analogous tridentate [C,N,S] ligands with lithium tetrachloropalladate gave the corresponding mono‐nuclear palladacycles. The ensuing phosphine derivatives could not be attained by direct reaction with the appropriate phosphine, but by pretreatment of the compound with silver perchlorate instead; this is due to the strong binding of tridentate ligand to the metal center, hindering the possibility of liberating palladium into the reaction media. Most of the compounds were applied to the Suzuki‐Miyaura cross coupling reaction between a phenylboronic acid and a conveniently substituted aryl bromide in either aqueous THF or EtOH, opening new routes towards a fully green cross coupling process. Those that gave good conversions contained the bidentate imine ligands, except for the triphenylphosphine species which rather surprisingly were scarcely active. The best results for compounds showing greater catalytic activity were **4 a** and **4 b** (bidentate [C,N] palladacycles) with chelated dppm. Notwithstanding, positive results although with rather low performances were also obtained with **2 c** and **2 e** (tridentate [C,N,S] palladacycles); furthermore, the results were better than using palladium (II) acetate under analogous conditions.

## Supplementary Materials

**Accession Codes**: CCDC 2368591 (**4 a**), CCDC 2368592 (**1 c**), and CCDC 2368593 (**e**) contain the supplementary crystallographic data for this paper. These data can be obtained free of charge via www.ccdc.cam.ac.uk/data_request/cif, or by emailing data_request@ccdc.cam.ac.uk, or by contacting The Cambridge Crystallographic Data Centre, 12 Union Road, Cambridge CB2 1EZ, UK; fax: +44 1223 336033.

## Author Contributions

Conceptualization, J.M.V. and F.R.; methodology, G.A.; software, F.R.; validation, J.M.V., B.a.J. and F.R.; formal analysis, J.M.O.; investigation J.M.V.; resources, J.M.O.; data curation, B.a.J.; writing—original draft preparation, J.M.V. and B.a.J.; writing—review and editing, J.M.V.; visualization, J.M.V. and F.R.; supervision, J.M.V.; project administration, J.M.O.; funding acquisition, J.M.O. All authors have read and agreed to the published version of the manuscript.

## Conflict of Interests

The authors declare no conflict of interest.

1

## Data Availability

Research data are not shared.
